# Dealing with Food and Eggs in Mouthbrooding Cichlids: Structural and Functional Trade-Offs in Fitness Related Traits

**DOI:** 10.1371/journal.pone.0031117

**Published:** 2012-02-14

**Authors:** Tim tkint, Erik Verheyen, Barbara De Kegel, Philippe Helsen, Dominique Adriaens

**Affiliations:** 1 Department of Biology, Research Group Evolutionary Morphology of Vertebrates, Ghent University, Ghent, Belgium; 2 Vertebrate Department, Royal Belgian Institute of Natural Sciences, Brussels, Belgium; 3 Department of Biology, Research Group Evolutionary Ecology, University of Antwerp, Antwerpen, Belgium; Biodiversity Insitute of Ontario - University of Guelph, Canada

## Abstract

**Background:**

As in any vertebrate, heads of fishes are densely packed with functions. These functions often impose conflicting mechanical demands resulting in trade-offs in the species-specific phenotype. When phenotypical traits are linked to gender-specific parental behavior, we expect sexual differences in these trade-offs. This study aims to use mouthbrooding cichlids as an example to test hypotheses on evolutionary trade-offs between intricately linked traits that affect different aspects of fitness. We focused on the oral apparatus, which is not only equipped with features used to feed and breathe, but is also used for the incubation of eggs. We used this approach to study mouthbrooding as part of an integrated functional system with diverging performance requirements and to explore gender-specific selective environments within a species.

**Methodology/Principal Findings:**

Because cichlids are morphologically very diverse, we hypothesize that the implications of the added constraint of mouthbrooding will primarily depend on the dominant mode of feeding of the studied species. To test this, we compared the trade-off for two maternal mouthbrooding cichlid species: a “suction feeder” (*Haplochromis piceatus*) and a “biter” (*H. fischeri*). The comparison of morphology and performance of both species revealed clear interspecific and intersex differences. Our observation that females have larger heads was interpreted as a possible consequence of the fact that in both the studied species mouthbrooding is done by females only. As hypothesized, the observed sexual dimorphism in head shape is inferred as being suboptimal for some aspects of the feeding performance in each of the studied species. Our comparison also demonstrated that the suction feeding species had smaller egg clutches and more elongated eggs.

**Conclusions/Significance:**

Our findings support the hypothesis that there is a trade-off between mouthbrooding and feeding performance in the two studied haplochromine cichlids, stressing the importance of including species-specific information at the gender level when addressing interspecific functional/morphological differences.

## Introduction

Mitochondrial DNA shows that the Lake Victoria super-flock of cichlids has given rise to more than 500 species in less than 200 000 years [1], [2]. This unusual high rate of speciation has given rise to numerous lineages that occupy almost every niche available in this young lake. Especially the diversity of trophic adaptations is remarkable, resulting in a wide range of phenotypes which are classified into trophic guilds [3].

As evolutionary processes are known to be constrained at different levels (e.g., [4], [5], [6], [7]), the evolutionary morphospace of the head region of these fishes can be expected to be highly constrained due to the integration of several components that impose different, sometimes conflicting functional demands (e.g. improved performance for speed is inversely related to performance for force, simply because of mechanical constraints). For cichlid species that represent different trophic guilds, these conflicts are especially apparent when the comparison involves a so-called ‘suction feeding’ species (i.e. a high velocity feeding method) and a so-called ‘biting species’ (i.e. feeding on hard prey). The differences between these very divergent modes of feeding are even apparent by the straightforward comparison of the components that make up the feeding apparatuses in these fishes, and the inference of their functional properties (for example the mechanical lever ratio's for lower jaw opening and closing in cichlid species characterized as ‘biters’ and ‘suckers’) [8]. However, because in most mouthbrooding cichlids, it is either only the female or the male parent that incubates the fertilized eggs in their buccal cavity, phenotypic differences (which may or may not be as large as species-specific differences), and therefore also adaptive peaks of a particular species may be sex-specific. It is obviously important to understand how sex-specific constraints may affect the ‘optimal design’ of a phenotype that combines various functions such as feeding, respiration and aspects related to reproductive behavior (mouthbrooding, nest building, agonistic display) [9], [10], [11], [12]. Earlier studies have shown that the combination of all these selective pressures yielded a series of morphologically similar cichlid species that repeatedly and independently evolved in the different African lakes [4], [13], [14], [15], [16]. As the shape of the head region of these mouthbrooding cichlid species is mostly defined by the shape and size of the buccal cavity, it can be expected that the evolutionary histories leading to the origin of these ecomorphs are the outcome of the combined, but different trade-offs between the spatial and functional demands imposed on the shape of jaws and related features for feeding and mouthbrooding. In addition, and depending on the gender that incubates the eggs, this difference may also have resulted in sexual dimorphism in the head-region, rather than being the result of sexual selection alone [17]. Besides requiring behavioral and physiological adaptations [3], [18] mouthbrooding has a negative impact on the number of offspring per reproductive effort. Despite this consequence, mouthbrooding might be one of the key innovations underlying the success of this group (next to pharyngeal jaw specializations [19], [20]). For a mouthbrooder, an increased buccal cavity volume offers several advantages: it increases the reproductive potential for a given egg size [21], it potentially improves the efficiency of oxygen uptake with a mouthful of eggs, and it provides the necessary water volume to churn the eggs for aeration [22]. The prediction that the mouthbrooding sex has a higher buccal volume, has been confirmed for paternal mouthbrooding cardinalfishes [21], [23] and a tilapiine cichlid [24], but its effect on feeding efficiency has not yet been established.

The observed diversity in trophic morphologies in cichlids is mainly reflected in the shape of the head-region (and hence of the buccal cavity) which reflects each species' ecomorphology [15], [25]. Although the overall morphological features of a functional ‘specialist’ species are not always unambiguously different from those in a species with a ‘generalist’ feeding repertoire [26], typical ‘suction feeders’ tend to have a more elongated and conical head shape, whereas ‘biters’ are often characterized by a shorter and wider head shape [27]. The corresponding spatial differences in lever systems and muscle organization result in a trophic apparatus that is either kinematically, or force efficient. In both cases, the head shape also determines the shape and size of the buccal cavity (as well as other components of the head that are relevant for mouthbrooding) [28], [29]. Obviously, the differences in the head shapes of ‘biting’ and sucking’ species may impose conflicting spatial demands, and may result in a situation where the requirements to optimize the feeding performance may be different or even opposite to the requirements maximizing mouthbrooding performance. It could be logical to assume that the space available for mouthbrooding will be more constrained in a ‘biting’ species with short jaws and large adductor muscles than in a ‘suction feeding’ species with less muscled long and slender jaws. Should this working hypothesis be correct, this trade-off between mouthbrooding and feeding performance might be an important factor to consider in the processes of morphological differentiation that occurred during the adaptive radiation of these haplochromine cichlids. Indeed, both functions are predicted to affect two very different aspects of these species' fitness: the number of offspring they can produce per litter and the efficiency by which they can process food to obtain the necessary amount of energy to live and reproduce.

It is the aim of this study to use theoretical capacities as proxies for both performances, by testing whether a difference in this trade-off exists in two mouthbrooding haplochromine species, with different mechanical requirements for feeding: one ‘suction feeding’ zooplanktivore species (velocity dependent prey capture) and a ‘biting’ molluscivore species (force dependent prey capture). *Haplochromis piceatus* Greenwood & Gee 1969 is used as a typical suction feeding species (ecomorph) with a long, pointed snout and elongate, gracile jaws. The diet of this zooplanktivorous species includes cladocerans, copepods, insect larvae and pupae [30], [31]. In contrast, *Haplochromis fischeri* Seegers 2008 ( = *Haplochromis sauvagei* non Pfeffer 1896: Greenwood 1980) is a typical biter with short and stout jaws. Its diet mainly consists of molluscs and only seasonally includes diatoms and copepods [32]. Unlike most other molluscivore haplochromines, this species does not ingest and crush the snail with its pharyngeal jaws, but extracts the snail from its shell by grabbing the exposed soft parts of its prey with its oral jaws, and shaking fiercely [33]. Both species are endemic to Lake Victoria and consequently diverged very recently from a common ancestral phenotype.

This study compares head shape data of both species using geometric morphometrics as well as morphological proxies for their feeding performance (through the inference of kinematic transmission efficiency for jaw protrusion and physiological cross section areas to estimate jaw muscle force). The quantified difference in these performance parameters in both species (ecomorphs) were used to test the following two hypotheses: (1) whether functionally relevant morphological features of ‘biting’ versus ‘suction feeding’ ecomorphs cause differences in the mouthbrooding performances of each species (estimated through the number, size and shape of eggs incubated in the buccal cavity), and (2) whether, as the result of gender specific differences related to mouthbrooding, females of both species are morphologically and functionally more constrained than males to perform tasks related to their respective trophic specialization as a ‘biter’ or a ‘suction feeder’. With respect to the first hypothesis, we predict a higher kinematic efficiency for upper jaw protrusion in the ‘suction feeding’ species (ecomorph), and a higher jaw muscle contraction force in the biter. We also expect to detect differences in the number, size and shape of the eggs that would agree with different strategies for efficient incubation and churning during mouthbrooding, in relation to the buccal cavity shape and size of both species (ecomorphs). With respect to the second hypothesis, we expect to observe smaller jaw muscles and a lower biting force in females of both, with the most important difference between males and females of the ‘biting’ species (ecomorph).

## Results

### Morphometric analysis

**Figure 1:**
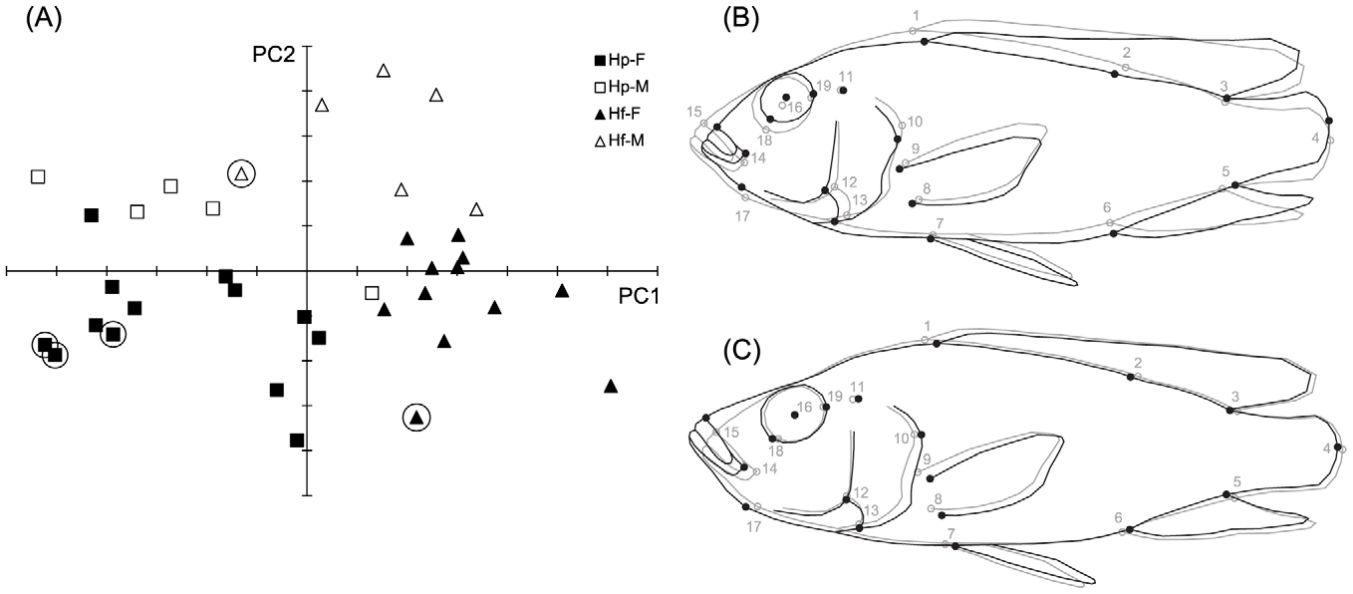
Body shape variation along the first two principal axes. (A) Plot of PC1 versus PC2 (explaining 44% and 17% of the variation, respectively) with indication of species and sex (Legend: Hp-F = *Haplochromis piceatus* females; Hp-M = *Haplochromis piceatus* males; Hf-F = *Haplochromis fischeri* females; Hf-M = *Haplochromis fischeri* males; wild caught specimens are circled). The warped outline drawings represent (B) the positive extreme of PC1 and (C) the negative extreme of PC2 (black outlines) compared to the consensus configuration (gray outline).

The geometric morphometric analysis of body shape (figure 1A) shows that overlap between species and sexes is limited. The wild-caught specimens of *H. piceatus* and *H. fischeri* also clustered within the corresponding range of both species.

Differences between the two species are reflected by PC1, whereas sexual differences are represented by PC2. The positive PC1 scores for *H. fischeri* reflect a relatively shortened head, with shorter jaws and a more rostrally positioned opercular region (with respect to the consensus) (figure 1B). The eye is shifted dorsally, resulting in a more rounded head profile, and eye diameter is smaller. The anal fin is longer.

The distribution of males and females on PC 2 indicates that similar sexual dimorphism is present for both species. Females tend to have a longer head, which is mainly due to a more rostral and dorsal positioning of the jaws, and an enlargement of the opercular region, without much change in actual length of the oral jaws (figure 1C).

Evaluation of group differences with a permutation test based on Squared Mahalanobis distances showed that both species and the sexes within each species occupied significantly different regions of the morphospace (all p-values for pairwise comparisons <0.002). Measurement of snout width on the dorsal pictures showed that *H. fischeri* had a broader snout than *H. piceatus* (F_1,15_ = 7.12, p = 0.018). Buccal volume as approximated by elliptical cylinders was equivalent for both species (F_1,14_ = 0.17, p = 0.689) but differed significantly between sexes (F_1,14_ = 7.32, p = 0.017) with females having larger buccal cavities than males.

### Feeding Performance

**Figure 2:**
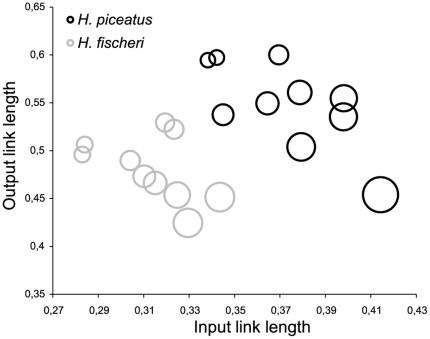
Plot of input link length (lower jaw coronoid processus) versus output link length (maxilla). The size of the circles indicates the value of KT.

Muscle mass of all three parts of the adductor mandibulae differed significantly between species even when standardized for head length, with *H. piceatus* having the lowest relative masses (A_1_: F_1,15_ = 32.51, p<0.0001, A_2_: F_1,15_ = 54.68, p<0.0001, A_3_: F_1,15_ = 25.40, p<0.0001) (Table 1). For the parts A_2_ and A_3_ of the adductor mandibulae, males also had significantly larger muscles than females (A_2_: F_1,15_ = 7.38, p = 0.016, A_3_: F_1,15_ = 8.41 p = 0.011). Species and sex also had a significant effect on standardized theoretical bite force exerted by A_2_ and A_3_ (A_2_: species: F_1,15_ = 69.45, p<0.0001, sex: F_1,15_ = 6.04, p = 0.027, A_3_: species F_1,15_ = 32.01, p<0.0001, sex: F_1,15_ = 8.94, p = 0.009). *Haplochromis fischeri* bites relatively harder than *H. piceatus*, and males bite harder than females. As a consequence total bite force, which is the sum of bite forces exerted by A_2_ and A_3_, follows the same pattern. As can be seen from table 1 most factors in the bite model (PCSA, MA and σ) contributed to the significant difference in bite force. Kinematic transmission coefficients of the anterior jaws did not differ between species (F_1,16_ = 0.15, p = 0.71), although the relative length of the input and output link of the system seems to be shorter for *H. fischeri* (figure 2). Females also had higher KT values than males (F_1,16_ = 8.50, p = 0.01). *Haplochromis fischeri* did have a significantly lower kinematic efficiency for the opening lever of the lower jaw (F_1,16_ = 34.56, p<0.0001). Both parameters of the upper jaw (relative length of the ascending process of the premaxilla (F_1,15_ = 29.75, p<0.0001) and angle between ascending and dentigerous process (F_1,15_ = 77.82, p<0.0001)) differed between species, where *H. piceatus* had a longer ascending process and a more acutely angled premaxilla.

**Table 1:** Metric data on the jaw muscles and estimates of feeding performance.

	*H. piceatus*		*H. fischeri*		Main-effect	
	Female (n = 5)	Male (n = 5)	Female (n = 5)	Male (n = 5)	species	sex
**Muscle mass (g)**						
Pars A1	15.670±4.328	22.320±1.882	39.920±6.847	67.280±19.776	F_1,15_ = 32.5***	F_1,15_ = 2.4
Pars A2	8.940±2.809	15.260±1.984	27.450±4.403	46.180±11.525	F_1,15_ = 54.7***	F_1,15_ = 7.4*
Pars A3	2.490±0.891	4.420±0.665	6.110±1.078	11.170±2.886	F_1,15_ = 25.4***	F_1,15_ = 8.4*
**Pars A2**						
Fiber length (mm)	4.090±0.629	5.251±0.437	4.579±0.514	5.026±0.275	F_1,15_ = 1.4	F_1,15_ = 2.4
PCSA (mm^2^)	0.022±0.007	0.029±0.002	0.061±0.013	0.091±0.019	F_1,15_ = 38.5***	F_1,15_ = 1.8
MA	0.394±0.025	0.424±0.021	0.484±0.045	0.559±0.043	F_1,16_ = 51.5***	F_1,16_ = 11.0**
σ (°)	50.372±2.390	49.962±4.190	54.153±2.639	56.304±2.754	F_1,16_ = 13.6**	F_1,16_ = 0.4
Bite force (N)	0.125±0.034	0.178±0.019	0.454±0.108	0.806±0.191	F_1,15_ = 69.5***	F_1,15_ = 6.0*
**Pars A3**						
Fiber length (mm)	3.829±0.651	4.600±0.839	4.555±0.307	5.172±0.981	F_1,15_ = 0.1	F_1,15_ = 0.0
PCSA (mm^2^)	0.006±0.001	0.010±0.002	0.013±0.002	0.022±0.004	F_1,15_ = 31.5***	F_1,15_ = 8.9**
MA	0.249±0.009	0.274±0.010	0.314±0.017	0.297±0.022	F_1,16_ = 39.4***	F_1,16_ = 0.3
σ (°)	34.444±6.824	30.377±4.417	29.397±2.671	36.512±5.645	F_1,16_ = 0.06	F_1,16_ = 0.4
Bite force (N)	0.017±0.004	0.025±0.004	0.039±0.009	0.072±0.019	F_1,15_ = 32.0***	F_1,15_ = 8.9**
**Total bite force**	0.142±0.033	0.204±0.022	0.493±0.115	0.878±0.208	F_1,15_ = 73.8***	F_1,15_ = 7.0*
**KT**	0.758±0.090	0.607±0.044	0.691±0.076	0.649±0.079	F_1,16_ = 0.2	F_1,16_ = 8.5*
**KE**	5.379±0.717	5.214±0.576	3.653±0.306	3.992±0.563	F_1,16_ = 34.6***	F_1,16_ = 0.1
**ASC/HL**	0.395±0.020	0.360±0.020	0.305±0.036	0.316±0.025	F_1,15_ = 29.8***	F_1,15_ = 1.0
**β (°)**	77.433±4.337	77.914±4.275	93.671±5.577	99.217±4.051	F_1,15_ = 77.8***	F_1,15_ = 2.0

**PCSA** = Physiological cross-sectional area; **MA** = mechanical advantage; **σ** = insertion angle; **KT** = kinematic transmission coefficient; **KE** = Kinematic efficiency of jaw opening; **ASC/HL** = ratio of the length of the ascending arm of the premaxilla and head length; **β** = angle between ascending and dentigerous arm of the premaxilla. * p<0.05, ** p<0.01, *** p<0.001.

Values are mean ± standard deviation.

### Egg parameters

Egg size did not differ between species (area: t_19_ = −0.19, p = 0.84, diameter: t_19_ = 1.56, p = 0.14, egg volume: t_19_ = −0.86, p = 0.40), but we did find a highly significant difference in egg shape (Table 2). Eggs of *H. piceatus* had a significantly higher aspect ratio (t_19_ = 6.83, p<0.0001). A Poisson generalized model showed that *H. piceatus* had smaller clutches, but this difference was only marginally significant after standardization for HL (χ^2^_(df = 1)_ = 3.60, p = 0.0579). The difference in calculated brood volume also became non-significant after standardization (F_2,18_ = 3.91, p = 0.0636).

**Table 2:** Egg measurements.

	*H. piceatus* (n = 10)	*H. fischeri* (n = 11)
Clutch size (# eggs)	33±10	57±20
Aspect ratio	1.37±0.03	1.28±0.03
Area (mm^2^)	5.20±0.64	5.21±0.25
Maximum diameter (mm)	3.07±0.21	2.96±0.08
Egg volume (mm^3^)	6.94±0.86	6.95±0.33
Brood volume (mm^3^)	227.5±82.2	397.3±139.0

Values are mean ± standard deviation.

## Discussion

### Structural and functional characterization of the biter versus the sucker

The observed species-specific head shape variation is in accordance with Barel's [27] description of the dichotomy between ‘biters’ and ‘suckers’ and with Cooper & Westneat's [34] findings on the morphological differentiation between damselfish herbivores and zooplanktivores. The biter (*H. fischeri*) has a shorter head and a more obtuse head profile, largely due to a shortening of the jaws (figure 1B). Such a shortening has the clear advantage of improving the force transmission (MA) of the jaw, when the input links remain the same. The accommodation of the significantly larger jaw adductors within the head of *H. fischeri*, seems to be associated with the head being broader, whereas the eye is shifted dorsally and reduced in diameter. Albertson & Kocher [8] reported a similar dorsal shift of the eye, associated with a dorsal expansion of the A_1_ part of the adductor mandibulae (lying ventral to the eye) for *Labeotropheus fuelleborni* (a Lake Malawi cichlid species with a biter morphotype). Through computer modeling, Otten [35] predicted that such a dorsal shift of the eye in cichlids not only increases bite force by providing more space for the muscles, it also allows a more favorable insertion of the A_1_ onto the maxilla improving force transmission to the jaws. Furthermore, our observations support the model predictions that a shorter ascending process of the premaxilla placed at an obtuse angle to the dentigerous arm improves force transmission, as this was indeed the case for *H. fischeri*.

The observed morphological differences between the species clearly represent difference in the feeding performance for ‘biting’ versus ‘sucking’. The estimated largest bite forces in *H. fischeri* are not only achieved by an increase in muscle mass, which is a plastic trait that can also be induced by feeding on hard food items [36], but also resulted from an improved force transmission (MA) of the lower jaw and a more favorable insertion (σ) of the jaw adductors.

Although KT is a good predictor of jaw protrusion [37], [38], [39] and zooplanktivores often have a higher KT than other trophic groups [40], we found no significant difference between *H. piceatus* and *H. fischeri*. This could reflect the fact that neither of the two species is considered as the most specialized within its trophic guild [41]. However, similar KT-values of both species are obtained in different ways (figure 2). The shorter links for *H. fischeri* improve force transmission, but also reduce the extent of jaw protrusion, making suction performance more expensive [27]. As a result of the functional redundancy in the four-bar system involved, different morphologies can result in similar KT values. In fact, the observed KT values fall within the range that has the most theoretically possible morphological solutions [42]. This allowed *H. fischeri* to have an oral jaw system that is more efficient in force transmission, due to its shorter jaws, without compromising its KT. It could be hypothesized that similar KT values in ‘biters’ and ‘suckers’ reflect a selective pressure constraining protrusion performance (independent of the morphological configuration to achieve this), similar to that of the ancestral condition of both species (considering their recent common ancestry). However, further comparative studies supported with detailed phylogenetic divergence estimates are required to test this properly.

### Sexual dimorphism and possible trade-offs in mouthbrooding females

As traits linked to the buccal cavity in female haplochromine cichlids are related to the efficiency for respiration, feeding and mouthbrooding, it can be expected that sexual dimorphism in the head is due to the added constraint of optimizing buccal incubation (mouthbrooding) performance. Although other aspects undoubtedly play a role as well (e.g. male territorial fighting), the most striking changes we observed result in an increased relative size of the buccal cavity in females. For example, the enlargement of the suspensorial compartment that increases the available volume of the buccal cavity in females is achieved by a longer snout, without much change in length of the jaws. In addition, we observed an enlargement of the opercular compartment of the head by a posterior shift of the base of the pectoral (and pelvic) fins and by a ventral displacement of the interopercle. Interestingly, similar expansions of the lateral aspect of the buccal cavity has also been reported for females of different *Tropheus sp.* populations, a female mouthbrooding cichlid genus that is endemic to Lake Tanganyika [43].

Although the relative size of the buccal cavity did not differ between species, we did observe a trend in clutch size: *Haplochromis piceatus* seemed to have fewer eggs in a buccal cavity of the same size. The eggs of H. piceatus also had a higher aspect ratio, which allows a more efficient packing with less jamming [44], so we might expect that it is easier for this species to churn the eggs in the mouth for aeration. For cardinalfishes it has indeed been found that such a reduction in brood size improves hypoxia tolerance [18]. Physiological performance testing of respiration, in combination with using buccal casts [21], [23], would allow a more direct quantification of the trade-off between respiration, feeding and mouthbrooding in females.

Although not all morphological differences between both sexes are statistically significant, most of the determined feeding characteristics suggest that for both species males are better ‘biters’ than females. Although our model for bite force calculation excluded the A_1_ part of the musculus adductor mandibulae (more than 50% of adductor mass) and didn't take the possible sexual difference in muscle physiology into account [45], we found several morphological indicators that support our hypothesis that bite force is higher in males then in females. This does, however, not necessarily imply that reduced theoretical bite performance in females is the exclusive result of an evolutionary trade-off between biting and mouthbrooding capacity. Indeed, as courtship behavior in haplochromine males involves defending a territory [46], it remains possible that higher bite forces in males are the result of other selection factors. Despite the fact that both studied species seem to be typical for their morphotype, a broader species sampling would allow us to determine if the observed patterns in mouthbrooding and feeding performance can be generalized to other species of the same morphotype.

### Conclusions

By studying a biting and a sucking ecomorph within haplochromine cichlids, we have shown that observed differences in the head morphology reflect functional demands related to the trophic guild to which they belong. Our data support the hypothesis that the sexual dimorphism in the head region involves an enlargement of the buccal cavity in females to brood eggs, but that this is not without consequences for feeding performance (e.g. bite force). As such, our findings support the hypothesis that a trade-off exists between functional performances that indirectly (feeding) and directly (mouthbrooding) influence fitness in the two species studied. It also suggests that the vast range of selective environments that arose during the explosive radiations of African Lake cichlids may need to be considered at the sex level, rather than the species level in mouthbrooding species. However, a more comprehensive survey in multiple lineages would be required to confirm this.

## Materials and Methods

### Specimen collection

The specimens used for this study came from laboratory reared stocks at the Royal Belgian Institute of Natural Sciences (Brussels). These stocks are derived from animals caught in the wild during the 1980's and have been tank bred for approximately 30 generations. The animals were killed with an overdose of MS-222 (*H. piceatus* ♀ n = 10, ♂ n = 5; *H. fischeri* ♀ n = 11, ♂ n = 5). All specimens were sexually mature and females were sacrificed during mouthbrooding. The standard length of the specimens ranged from 57 to 87 mm for *H. piceatus* and 76 to 114 mm for *H. fischeri*. After fixation in 10% formalin for at least two weeks the specimens were transferred to 70% ethanol for preservation. In accordance with the Belgian national law concerning the protection and wellbeing of animals of August 14, 1986, a formal approval from an ethical committee is not required for this kind of project.

As the morphology of tank bred cichlids is known to be variable and might be different from animals in the wild [47], we also included some wild caught specimens, which were provided by the National Natural History Museum (Leiden, The Netherlands) (*H. piceatus*: RMNH 62769 (n = 3) and *H. fischeri*: RMNH 70426 (n = 2)).

### Biometry

The left side of all specimens was photographed using a Nikon D40x digital reflex camera, equipped with the standard zoom-nikkor 18–55 mm lens. The fishes were pinned in a dissection board to standardize orientation and spread out the fins. In every picture an individual code and a scale marker was included to allow identification and scaling. Head length (HL) of every specimen was measured on these pictures as defined in Barel et al. [48]. The eggs of the mouthbrooding females were extracted and photographed with a digital camera (Colorview 8, Soft Imaging System) mounted on a dissection microscope (Olympus SZX9). The shape of the eggs was analyzed by taking the following measurements: the length of the long and short axis (assuming it were ellipses), aspect ratio and area. The volume of the eggs was approximated by assuming they were ellipsoids with a long axis and two equal short axes.

For each species five females and five males were dissected to extract the muscles operating the oral jaws. For each individual the A_1_, A_2_ and A_3_ parts of the adductor mandibulae complex were extracted and weighed on an electronic balance (Sartorius BP 121S) to the nearest 0.1 mg (For details on jaw adductor muscle anatomy see [49]). During the dissections photographs were made (with a Nikon D40x and a Sigma 150 mm macro lens) to document the attachment of the muscles to the head skeleton. Snout width was determined on a dorsal picture of these dissected specimens.

The volume of the buccal cavity was approximated using a series of elliptical cylinders as proposed by Drost and Van den Boogaart [50] (see also [51], [52]). We measured the width and height of the buccal cavity, which equal the major and minor axis of these ellipses, at 100 equally spaced intervals. These measurements were taken from lateral and ventral pictures of the dissected specimens after clearing and staining [53].

### Geometric Morphometrics

**Figure 3:**
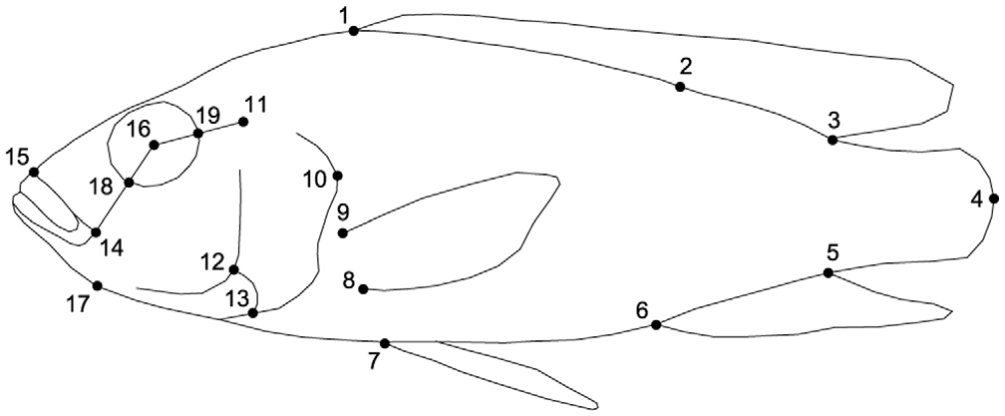
Outline drawing with indication of landmark positions. (1) Anterior insertion of the dorsal fin (2) Insertions of the most caudal spiny fin ray of the dorsal fin (3) Posterior insertion of the dorsal fin (4) Posterior end of the lateral line (5) Posterior insertion of the anal fin (6) Anterior insertion of the anal fin (7) Insertion of the leading edge of the pelvic fin (8) Insertion of the trailing edge of the pectoral fin (9) Insertion of the leading edge of the pectoral fin (10) Posterior extremity of the operculum (11) Center of neurocranial lateral line foramen 5 (12) Dorsal intersection of subopercle and interopercle (13) Ventral intersection of subopercle and interopercle (14) Posterior extremity of the gape (15) Intersection between upper lip and body outline (16) Center of the eye (17) Retroarticular process (18) Intersection of the line connecting landmarks 14 and 16 and the eye outline (19) Intersection of the line connecting landmarks 11 and 16 and the eye outline.

The coordinates of 19 landmarks were determined on the habitus pictures of the left side (figure 3) using TPSDIG 2 [54]. Measuring error resulting from variation in positioning of the specimens for photographing and in digitizing of landmarks was quantified, based on two specimens per sex per species (following protocol of Adriaens - http://www.fun-morph.ugent.be/Miscel/Methodology/Morphometrics.pdf). We found that 3.6% of the variation is due to digitization error and 25.4% is due to the combination of orientation and digitization error.

Non-shape variation was removed by performing a Generalized Procrustes Analysis, removing effects of size, position and orientation [55]. To allow the use of traditional multivariate techniques it is also necessary to project the shapes from the non-Euclidean shape space onto a Euclidean tangent space [56]. The correlation between the shape distances in both spaces was checked with TPSSMALL [57] and showed a perfect correlation (r = 1.0, slope = 0.9997). Shape variation was analyzed with a PCA using the coordinate data in MorphoJ 1.01a [58]. Due to the limited and unequal sample size we used a permutation test of Squared Mahalanobis distances (10 000 replicates) to test the significance of group differences.

### Bite model

The theoretical bite force exerted by the different parts of the adductor mandibulae was estimated using a static bite force model [59]. The output force at the jaw tip was calculated taking into account the maximal force produced by the muscle (based on the physiological cross-sectional area (PCSA) and an estimated unit contraction force – see below) and the geometry of the jaw.

After weighing the muscles (for volume calculation), the average fiber length was determined by immersing the bundles in 30% nitric acid (HNO_3_) to dissolve connective tissue holding the muscle fibers together [59]. After about 20 h (depending on muscle size) individual fibers were teased apart and the nitric acid reaction was stopped with an excess of saturated Borax solution (disodium tetraborate). The length of 30 individual fibers per muscle was measured on digital images using analySIS® software (Soft Imaging System) and average fiber length calculated. Muscle density was assumed to be 1 g.cm^−3^ [60], and unit contraction force 19 N.cm^−2^ [61] The contraction force produced by the muscle along its line of action (F_in_) can then be calculated as: F_in_ = PCSA*19 N.cm^−2^, where PCSA equals muscle volume divided by average fiber length.

Taking into account the orientation of the line of action of the muscle and the efficiency of force transmission by the lower jaw system, the output force at the jaw tip (F_out_) was calculated as follows: F_out_ = F_in_ * sin σ * L_in_/L_out_. The inlever (L_in_) is the distance between the articulation of the lower jaw with the quadrate and the insertion of the muscle onto the lower jaw. Likewise, the outlever (L_out_) was taken as the distance from the articulation to the jaw tip. The ratio of L_in_ to L_out_ then reflects the mechanical advantage for jaw closing. The σ reflects the angle between the line of action of the muscle and the inlever. All distances and angles were calculated based on the coordinates of four points (jaw tip, jaw articulation with the quadrate, muscle insertion on the jaw and muscle origin) determined on the photos taken during the dissection of the muscles. All calculations from coordinates and muscle fiber lengths to output forces were done in Excel® (Microsoft corporation). We determined the bite force exerted by the A_2_ and A_3_ part of the adductor mandibulae at an arbitrary gape angle of 20°, the A_1_ part was excluded due to its complex pennation and attachment to both the premaxilla and the lower jaw. Consequently, the obtained values are an underestimation of bite force for both species, but still allow meaningful comparison.

### Kinematic efficiency

As a measure for efficiency of suction feeding the kinematic transmission coefficient (KT) of the anterior-jaw four-bar linkage was calculated. In cichlids this system consists of the suspensorium as the fixed link, the nasal as coupler, maxilla as output link and coronoid portion of the lower jaw as input link [39]. This linkage describes the amount of maxillary rotation as a result of lower jaw depression, where the KT of this system is defined as the output rotation of the maxilla divided by the input rotation of the lower jaw. Calculations of this coefficient were based on the coordinates of the joints of the linkage determined on the dissection photos and were implemented in Excel® (Microsoft Corporation).

### Lever systems and force transmission

The mandible of fishes can be considered as a lever system rotating around the quadrate-articular joint. The outlever, which is the same for jaw opening and closing, is determined as the distance between the joint and the tip of the mandible. The inlever for jaw opening is the bar running from the joint to the tip of the retroarticular process (onto which the interopercular-mandibular ligament attaches). As suction feeding fish rely on fast jaw opening, the kinematic efficiency of this system was calculated as the ratio of outlever to inlever (for jaw opening). Higher values of this ratio represent a kinematically efficient system that more effectively amplifies the input velocity at the retro-articular process. Furthermore two characteristics were quantified that influence force transmission by the upper jaw: the relative length of the ascending process of the premaxilla (for a given head length) and the angle between the ascending and dentigerous arm of the premaxilla [35].

### Statistical analyses

Differences in egg metrics between species were analyzed with a t-test. Egg counts were compared using a Poisson generalized model with a log link function [62]. Differences in estimates of feeding performance between species and sexes were statistically evaluated with a glm implementation of a two-way ANOVA with inclusion of HL as covariate for variables that are size-related. All statistical analyses were performed using SAS 9.2.

## Acknowledgments

We wish to thank Frans Witte from the National Natural History Museum (Leiden, The Netherlands) for providing the wild-caught specimens and two anonymous reviewers for their suggestions to improve this manuscript.
